# Comparative Evaluation of ELISA Tests for Bovine Tuberculosis Detection: Implications for Improved Disease Control

**DOI:** 10.1155/tbed/5527662

**Published:** 2026-02-25

**Authors:** Carlos Eugênio S. Vidal, Ingrid Ieda F. Souza, Carlos Alberto N. Ramos, Jéssica F. Camargo, Cristina P. Araújo, Ana Luiza A. R. Osório, Denis A. Spricigo, Felipe Libardoni, Flábio R. Araújo, Cynthia Mantovani, Agueda C. Vargas

**Affiliations:** ^1^ Regional Technical Unit for Agriculture and Livestock of Santa Maria, Ministry of Agriculture and Livestock, Santa Maria, Rio Grande do Sul, Brazil, agricultura.gov.br; ^2^ Embrapa Beef Cattle, Campo Grande, Mato Grosso do Sul, Brazil; ^3^ Department of Veterinary Medicine, Federal University of Mato Grosso do Sul, Campo Grande, Mato Grosso do Sul, Brazil, ufms.br; ^4^ Department of Veterinary Medicine, Federal University of Rio Grande do Sul, Porto Alegre, Rio Grande do Sul, Brazil, ufrgs.br; ^5^ Zoonosis Control Center, Municipal Health Secretariat of Campo Grande, Campo Grande, Mato Grosso do Sul, Brazil; ^6^ Federal Agricultural Defense Laboratory, Porto Alegre, Rio Grande do Sul, Brazil; ^7^ Department of Preventive Veterinary Medicine, Federal University of Santa Maria, Santa Maria, Rio Grande do Sul, Brazil, ufsm.br

**Keywords:** bovine tuberculosis, diagnosis, diagnostic methods, ESAT-6, MPB70, MPB83

## Abstract

Accurate diagnosis of bovine tuberculosis (bTB) remains a critical challenge for disease control and eradication programs. While cell‐mediated immune (CMI) response tests such as the comparative cervical intradermal tuberculin test (CCITT) are widely used, their sensitivity is limited, especially in later stages of infection. Antibody‐based assays, such as enzyme‐linked immunosorbent assays (ELISAs), may offer complementary detection and improve case identification. This study aimed to comparatively evaluate two indirect ELISA tests for the detection of antibodies against *Mycobacterium bovis* in naturally infected cattle from Southern Brazil. One ELISA test is in an experimental phase and uses a chimeric antigen comprising fragments of MPB70, MPB83, and ESAT‐6 proteins. The second is a commercially available ELISA registered with the WOAH, based on MPB70 and MPB83 antigens. Serum samples were collected from 147 cattle across nine herds with known bTB epidemiological histories. All animals underwent CCITT, and a subset was subjected to post‐mortem examination, culture, and nested‐PCR. The experimental ELISA identified 16.6% of animals as seropositive, while the commercial ELISA identified 13.0%, both exceeding the 4.4% apparent prevalence detected by CCITT. Both ELISAs showed low sensitivity (<30%) when compared to culture or PCR‐confirmed cases but were able to detect additional infected animals missed by CCITT. These findings support the use of serological tests as complementary tools to enhance bTB detection in cattle and inform surveillance and eradication strategies in endemic regions.

## 1. Introduction

Bovine tuberculosis (bTB), caused by *Mycobacterium bovis*, is a chronic zoonotic disease of global significance, affecting livestock, wildlife, and humans. Economically, it leads to substantial losses due to reduced productivity, carcass condemnation, trade limitations, and the high costs of testing and culling. In low‐ and middle‐income countries, bTB continues to threaten food security and rural development, while in high‐income nations, it remains a persistent burden despite long‐standing control efforts. Public health concerns arise particularly in regions with limited pasteurization of dairy products or close human–animal interactions [1, 2].

In Brazil, the national strategy for bTB control is implemented through the National Program for the Control and Eradication of Animal Brucellosis and Tuberculosis (PNCEBT), which primarily employs a test‐and‐slaughter policy based on the comparative cervical intradermal tuberculin test (CCITT). Within this framework, a farm is designated as bTB accredited‐free under the PNCEBT by achieving two consecutive negative whole‐herd tests, conducted on all susceptible animals at intervals of 6–12 months. To maintain this crucial status, certified farms must continue to present negative whole‐herd tests annually. The program also imposes strict biosecurity measures, including conditions for the introduction of new animals, which must originate from other accredited‐free properties or pass two diagnostic tests with an intervening period of isolation. The loss of this status is also regulated: the detection of a bTB focus results in the temporary suspension of the certificate, requiring a sanitary culling of the infected animals and two subsequent negative whole‐herd tests before the accreditation can be reinstated [3].

The CCITT, which serves as the main diagnostic tool in these control programs, including the PNCEBT, targets the cell‐mediated immune (CMI) response, which generally dominates during the early stages of infection. However, the immunological landscape in infected cattle is complex. In many cases, humoral responses, including detectable antibody levels, may appear concurrently with or soon after the CMI response. Rather than reflecting a strict progression from CMI to antibody dominance, the variability in antibody detection often results from limitations in test sensitivity, particularly in the early phases of infection or in low‐titer animals [4, 5].

Serological assays, particularly enzyme‐linked immunosorbent assays (ELISAs), have been explored as complementary tools to improve the detection of infected animals. Their utility lies in their capacity for high‐throughput screening and their potential to detect animals missed by intradermal testing. Recombinant antigens, such as MPB70 and MPB83—highly expressed secreted proteins of *M. bovis*—and ESAT‐6—an early‐expressed virulence factor—have been used individually or in combination to enhance ELISA sensitivity [6–8]. These antigens elicit antibody responses at varying stages of infection, but clear patterns are difficult to generalize under field conditions. Furthermore, although animals with advanced infections may occasionally yield negative CMI responses, current evidence suggests that such animals are not necessarily the primary contributors to ongoing transmission within herds [9, 10].

In the absence of a true gold standard for bTB diagnosis, the performance of new serological tests is often compared relative to the CCITT. However, this comparison is constrained by the limitations of the CCITT itself, including imperfect sensitivity and specificity. As a result, conventional validation metrics may either underestimate or overestimate the utility of complementary diagnostic methods. In this context, agreement analysis and herd‐level stratification offer alternative means to assess test performance in endemic scenarios.

This study aimed to evaluate the diagnostic performance of an experimental indirect ELISA based on a chimeric fusion protein composed of MPB70, MPB83, and ESAT‐6. Its performance was compared to that of a commercially available ELISA registered with the World Organisation for Animal Health [11]. Both tests were applied to serum samples from naturally infected cattle belonging to herds with diverse infection histories and official bTB certification statuses. Results were interpreted in relation to CCITT outcomes and herd epidemiological context, with the goal of identifying potential advantages and limitations of each serological approach.

## 2. Materials and Methods

### 2.1. Study Design and Herd Selection

A cross‐sectional study was conducted on 21 cattle breeding farms located in five sub‐regions of Rio Grande do Sul, Brazil, as defined by Marvulo et al. [12]. The herds included 19 dairy and 2 beef operations (herds M and O). For the purpose of evaluating diagnostic accuracy, post‐mortem confirmation, and comparison between serological and skin tests, a subset of 147 animals from nine herds was followed through slaughter. These herds included both CCITT‐positive and bTB accredited‐free herds.

Herds were selected via convenience sampling to include farms with varying tuberculosis statuses: some had animals reactive to bTB diagnostic tests, and others were officially bTB accredited‐free (*n* = 6) under the Brazilian PNCEBT. Cattle over 6 weeks of age were tested by CCITT, excluding females within 15 days pre‐ or post‐calving. Over 3 years, more than 3000 animals were evaluated, but only data from the 147 cattle followed through to slaughter were used for sensitivity analysis.

While no formal statistical power analysis was conducted prior to sampling, the sample size was selected based on practical field considerations and aimed to include animals representing a range of infection statuses to maximize diagnostic evaluation accuracy.

### 2.2. CCITT

CCITT was performed by veterinarians authorized under the PNCEBT, following the guidelines published in 2017. The bovine and avian purified protein derivatives (PPDs) used in the skin test were sourced from Instituto Biológico, São Paulo, Brazil. These tuberculins conform to WOAH guidelines for potency testing and quality control, although no independent potency evaluation was performed during this study. The expected diagnostic sensitivity and specificity of the locally produced bovine PPD (bPPD), based on Brazilian field data, are approximately 80%–90% and >95%, respectively [13, 14].

Skin thickness was measured at both the bPPD and avian PPD (aPPD) injection sites. Results were interpreted based on the difference (Δ) between the two: reactive‐positive: Δ >4.0 mm; inconclusive: Δ >2.0 mm and <4.0 mm; negative: Δ <2.0 mm.

For analysis, inconclusive animals were considered negative, unless they had two consecutive inconclusive results, in which case they were reclassified as positive. Blood samples for ELISA testing were collected on the same day as the CCITT was performed.

### 2.3. Commercial ELISA

The IDEXX *M. bovis* antibody test kit was used according to manufacturer’s instructions. All serum samples were tested once (single well, no duplicates) with control samples run in duplicate. Results were expressed as the sample‐to‐positive (*S*/*P*) ratio. Plates were read at 450 nm using a SpectraMax M5 microplate reader (molecular devices), and *S*/*P* ratios were calculated in Excel as directed by the kit protocol.

### 2.4. Experimental Indirect ELISA

An in‐house indirect ELISA was developed using a chimeric antigen combining hydrophilic domains of ESAT‐6, MPB70, and MPB83 proteins [15]. Plates were coated with 0.2 µg/mL antigen (dilution 1:5000) in carbonate–bicarbonate buffer (pH 9.6) overnight at 4°C. After blocking with 5% skim milk in PBST, sera were diluted 1:600 and added in duplicate. Bound antibodies were detected with anti‐bovine IgG peroxidase conjugate (Sigma, A5295) at 1:10,000, followed by OPD substrate. Absorbance was read at 490 nm (Bio‐Tek EL‐800).

Intra‐test controls (duplicates) and inter‐test controls (positive and negative sera in triplicate) were used. The coefficient of variation (CV) threshold was 10%. Results were expressed as an *S*/*P* ratio, calculated by subtracting the negative control mean from the sample mean (*S*) and dividing by the difference between the positive and negative control means (*P*). The cutoff for positivity was herd‐specific and determined via scatterplots.

### 2.5. Post‐Mortem Sampling at Slaughter

Tissue samples were collected from 160 cattle at slaughter, of which 147 were retained for analysis due to proper identification. These animals belonged to nine herds, including the CCITT‐positive herd Q. Samples included medial retropharyngeal, tracheobronchial, and mesenteric lymph nodes, tonsils, and any gross lesions. Animals were classified as having visible bTB‐like lesions or not. Each tissue was split for microbiological culture and molecular analysis.

In the present study, animals were subjected to post‐mortem inspection, and any suspect lesions were collected for laboratory confirmation (culture and PCR). It is important to note that visible lesion detection at slaughter is an imperfect diagnostic method with variable sensitivity. Studies have shown that the overall surveillance sensitivity in abattoirs can be low (e.g., 31.4% in one low‐prevalence study), largely because a significant proportion of infected animals do not present macroscopically detectable lesions (MDL) [16]. Given these inherent limitations, detection of lesions was strictly complemented with culture and PCR confirmation to maximize the diagnostic accuracy.

### 2.6. Mycobacterial Culture and Microscopy

Tissues were homogenized with sterile sand and saline, filtered, centrifuged (1200 × *g*, 15 min), and decontaminated using Petroff’s method [17]. Sediments were plated on Stonebrink medium and incubated at 37°C for up to 90 days. Colonies were stained using the Ziehl–Neelsen method to detect acid‐fast bacilli (AFB). PCR was performed on all AFB‐positive cultures using primers JB21/JB22 targeting the *Mycobacterium tuberculosis* complex [18].

### 2.7. Direct Detection of *M. bovis* DNA in Tissues

For nested‐PCR, 100 mg tissue fragments at lesion borders were homogenized in PBS, and 200 µL used for DNA extraction with the DNEasy Blood & Tissue Kit (Qiagen). Nested‐PCR targeted TbD1 [19] and rv2807 [20]. In herd Q, histopathology and Gram/Ziehl–Neelsen stains were performed to validate positive PCR findings.

### 2.8. Data Analysis

All 147 animals were tested with both ELISA assays and the CCITT. Results near the cutoff were re‐tested. Receiver operating characteristic (ROC) curves were generated using MedCalc v9.6.4.0 to estimate sensitivity, specificity, AUC, and Youden’s index. Box plots and scatter plots were generated in Minitab v16.1.0.0 to visualize distribution by herd and test.

Two‐by‐two contingency tables were constructed in SAS, stratified by herd and test. The commercial ELISA served as reference for comparisons. Metrics included:

Apparent prevalence (Pa) = (*a* + *b*)/(*a* + *b* + *c* + *d*).

True prevalence (Pt) = (Pa + Sp – 1)/(Se + Sp – 1).

Kappa index, McNemar’s test, and odds ratios (all with 95% CIs).

Herd‐level bTB status was determined based on individual test results, following the herd classification approach by Martin et al. [21].

## 3. Results

### 3.1. Herd Background

Data were collected from 21 cattle herds, encompassing variables such as intervals between intradermal tuberculin tests, counts of negative, inconclusive, and positive test outcomes, total herd size at test date, and numbers of culled animals (Figure 1). Based on these data and CCITT results, herds were stratified into five epidemiological categories:

Figure 1Intervals between tests and the number of cattle diagnosed with bovine tuberculosis in each herd. (1) Three herds characterized as “unaccredited and lacking a history of infection” (A–C). (2) Three herds designated as “accredited free without a history of infection” (D–F). (3) Three herds identified as “accredited free with a history of infection” (G–I). (4) Two herds classified as “negative skin tests with a history of infection” (J and K). (5) Ten herds categorized as “positive skin tests with subsequent confirmation through positive PCR” (L–U). Italicized herds *M* and *O* = slaughter herds. Bold test number = collected for serology.  ^∗^Asterisk before and after = blood collected before and after tuberculin test application or reading, respectively. Positives and inconclusives refer to the tuberculin test.

**Figure:**
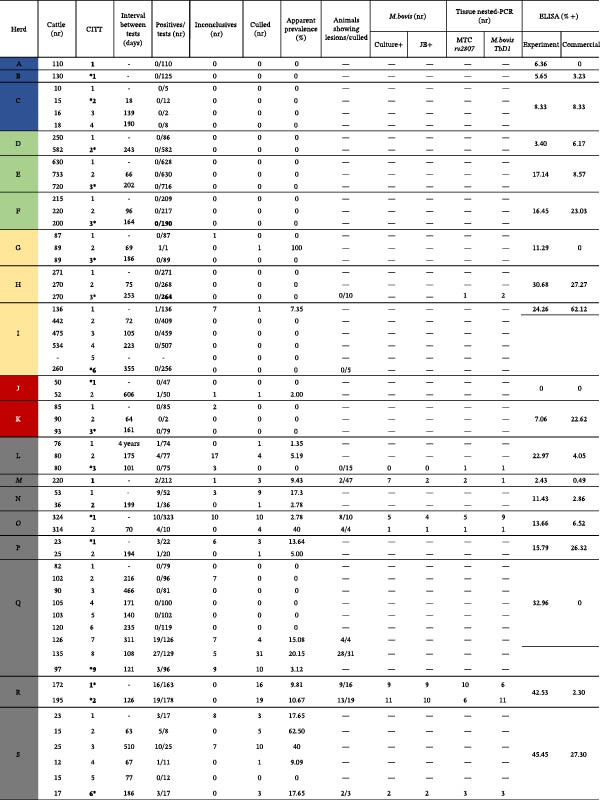
(A)

**Figure:**
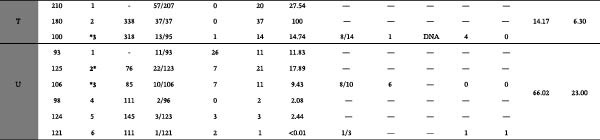
(B)

Unaccredited, no history of infection: herds A–C.

Accredited free, no history of infection: herds D–F.

Accredited free, with a history of infection: herds G–I.

Negative CCITT, with history of infection: herds J–K.

Positive CCITT, confirmed by PCR: herds L–U.

A notable case was herd L, where a repeatedly inconclusive animal by CCITT was sampled; subsequent PCR testing confirmed infection. This variation in serological responses underscores complexities in ELISA evaluation and suggests different infection control stages among herd categories.

### 3.2. Exploratory Analysis

To assess the diagnostic performance of the two ELISA tests relative to the current gold standard used in control programs, the CCITT, we conducted a ROC curve analysis. The ROC curves were used to compare the performance of the experimental and commercial ELISAs against the CCITT results, allowing us to evaluate potential cutoffs for classifying positive and negative animals. It is important to note that the negative grouping for the ROC analysis was defined broadly, including animals that tested negative on the CCITT across all herds, not just those restricted to officially accredited bTB‐free herds. This wider inclusion criterion should be taken into account, as it potentially impacts the specificity estimates derived from the ROC analysis.

**Figure 2:**
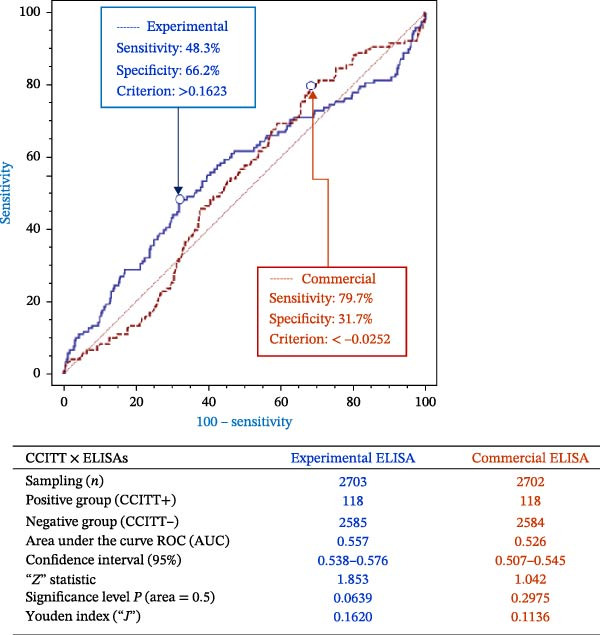
Receiver operating characteristic (ROC) curves of the experimental and commercial ELISA tests. AUC, areas under the curve.

ROC curve analysis compared ELISA results against CCITT as the reference standard (Figure 2). The negative and positive populations overlapped substantially, resulting in low sensitivity and specificity for both ELISAs (Youden’s index = 0.1620; agreement index = 0.1136). The experimental ELISA approached statistical significance at the 95% confidence level (*p*  = 0.0639), more so than the commercial ELISA (*p*  = 0.2975).

### 3.3. ELISA Results Per Herd

Box plot visualization of ELISA results per herd (Figure 3) demonstrated wider result dispersion for the experimental ELISA, applying the commercial ELISA cutoff of *S*/*p*  = 0.300. All herds except herd J had at least one animal exceeding this threshold by the experimental test. In contrast, herds A, G, J, and Q showed no positives with the commercial ELISA.

Figure 3ELISA results (*S*/*P* ratio) per herd in box plot graph. (A) Experimental. (B) Commercial. Cutoff points = 0.300.

**Figure:**
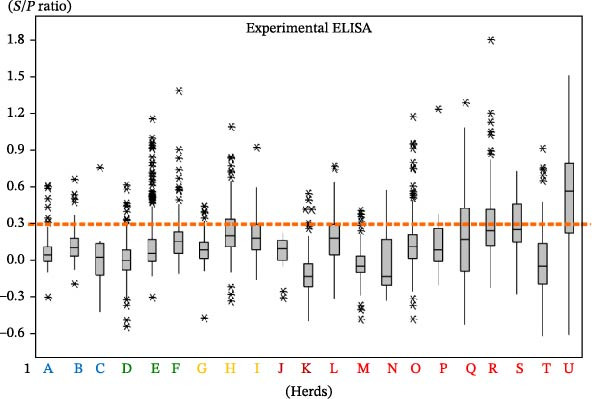
(A)

**Figure:**
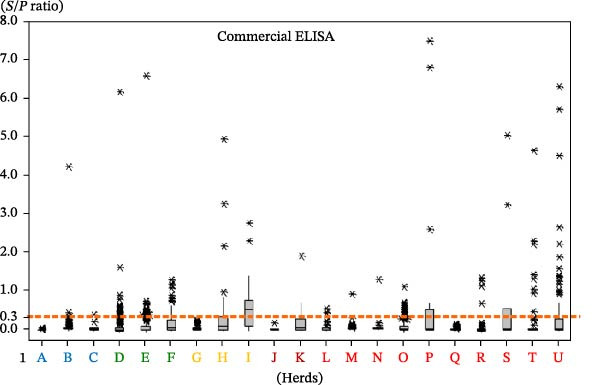
(B)

Herd J had limited sampling (19 of 50 animals), yet subsequently showed one CCITT‐positive animal 20 months later. Increasing the experimental ELISA cutoff to reduce false positives would reclassify herds G and M as negative. Herd M had confirmed infection via CCITT, lesions at slaughter, and microbiological confirmation. Herd G, although accredited free, included imported pregnant heifers with elevated *S*/*P* ratios and a CCITT‐positive animal upon introduction.

Applying the manufacturer’s commercial ELISA cutoff, herds A and Q would also be negative. This observation is significant because herd A had not undergone CCITT testing for over 2 years, posing a risk of undetected infection, while herd Q had previous CCITT‐positive results and confirmed cases by histopathology. Classifying herds with proven infection history as negative (e.g., herd Q) by the commercial cutoff highlights the potential for the commercial test to underestimate prevalence in field scenarios.

### 3.4. Distribution of Results Across Tests

**Figure 4:**
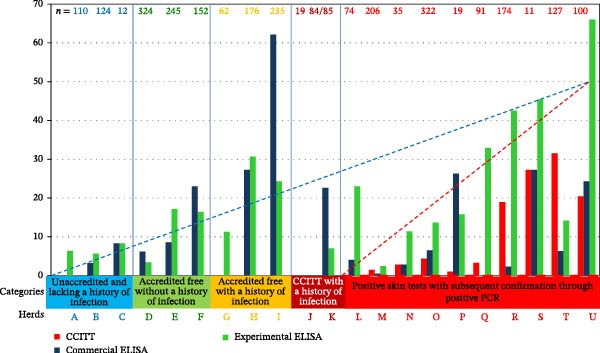
Positive results (%) in the 21 herds for the three different indirect tests in the comparative cervical intradermal tuberculin test (CCITT) 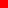, experimental ELISA 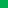, and commercial ELISA 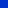. The number (*n*) of samples tested per herd and trendlines of humoral (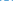) and cellular (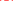) immune tests in the five categories in which the herds were grouped.

**Figure 5:**
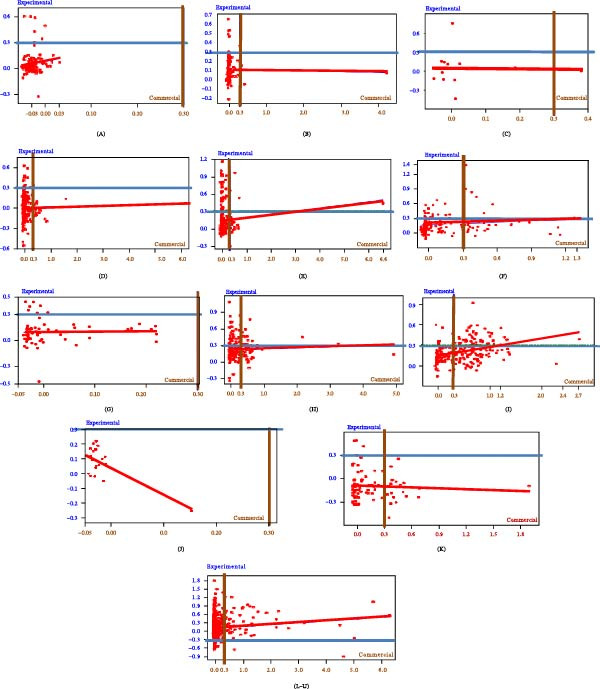
Scatter plot graphs of the commercial (*x*) versus the experimental (*y*) ELISA (*S*/*P* ratio) results by herds (from A to U) and grouped in lines. Cutoff values (0.300 for both ELISAs) are indicated by brown and blue lines as well as red trendlines for linear regressions.

Evaluating *S*/*P* ratios on a continuous scale allowed detailed interpretation beyond binary outcomes. The distribution of *S*/*P* ratios for both the experimental and commercial ELISAs is summarized in Figure 4. Scatter plots comparing ELISA results by herd revealed patterns of agreement and divergence (Figure 5).

Within unaccredited herds (A–C), a slightly lower cutoff than 0.300 for the experimental ELISA would classify an individual from herd A as positive. Commercial ELISA showed borderline values in herd B. The commercial test manufacturer recommends adjusting cutoffs based on herd epidemiology.

Among accredited free herds (D–F and G–I), the commercial ELISA detected more positives than in unaccredited herds, except for herd G, which was negative by commercial ELISA but had animals near cutoff and a known infection history.

In herds J and K (CCITT‐negative, infection history), the experimental ELISA detected positives in herd K, but not in Herd J. The *S*/*P* values in these herds were mostly under 0.6–0.7, mirroring some unaccredited herd results and suggesting a greater, albeit low‐level, seroreactivity detected by the experimental assay.

### 3.5. Applied Cutoff Threshold

Using a uniform cutoff of 0.300 *S*/*P* for both ELISAs, no herd showed 100% positivity in all tested animals, indicating lack of clear demarcation between infected and uninfected groups and highlighting the need for discriminant or latent class analyses instead of simple binary classification.

### 3.6. Herd Prevalence

Defining herd positivity as ≥1 positive animal at a 0.300 cutoff, the experimental ELISA detected positives in 20 of 21 herds (95.2%), commercial ELISA in 17 herds (80.9%), and CCITT in nine herds (42.8%). Only herd J was negative by both ELISAs. Seropositive animals were found by both ELISAs in all six accredited bTB‐free herds (6/6 and 5/6, respectively). Apparent and true prevalence estimates are shown in Table 1 (sections I.C and II.C).

**Table 1:** Diagnostic performance, agreement, and prevalence estimates of experimental and commercial ELISA tests in comparison with the comparative cervical intradermal tuberculin test (CCITT).

I. Experimental ELISA vs. CCITT			
ELISA result	CCITT positive	CCITT negative	Total
Positive	34	450	484
Negative	84	2135	2219
Total	118	2585	2703

II. Commercial ELISA vs. CCITT			
ELISA result	CCITT positive	CCITT negative	Total
Positive	11	352	363
Negative	107	2232	2339
Total	118	2584	2702

III. Experimental vs. commercial ELISA			
Result	Commercial positive	Commercial negative	Total
Experimental positive	110	374	484
Experimental negative	253	1965	2218
Total	363	2339	2702

*Note:* McNemar: 9.99 (*p*  = 0.002); general agreement: 80.24%; true prevalence (Pt): 4.36%. odds ratio: 1.92 (95% CI: 1.273–2.897); Kappa agreement: 4.60 (95% CI: 1.2–8). McNemar: 1.79 (*p*  = 0.180); general agreement: 83.01%; true prevalence (Pt): 4.36%. odds ratio: 0.65 (95% CI: 0.347–1.225); Kappa agreement: −2.16 (95% CI: −4.89–0.57). McNemar: 43.78 (*p*  = 0.000); general agreement: 76.79%; true prevalence (Pt): 13.42%. odds ratio: 2.28 (95% CI: 1.78–2.932); Kappa agreement: 12.55 (95% CI: 8.21–16.88).

Abbreviation: CI, confidence interval.

### 3.7. Agreement and Performance Measures

Kappa agreement among ELISAs and CCITT was minimal: 4.60% (experimental vs. commercial ELISA), −2.16% (commercial ELISA vs. CCITT), and 12.55% (experimental ELISA vs. CCITT) (Table 1). Sensitivities of the experimental and commercial ELISAs relative to CCITT were 28.81% and 9.32%, while specificities were 82.59% and 86.38%, respectively, with variability across herd categories. Detailed agreement indices, predictive values, and prevalence estimates for ELISA tests relative to CCITT and culture/PCR are presented in Table 2.

**Table 2:** Agreement, sensitivity, specificity, predictive values, and prevalence estimates of experimental and commercial ELISA tests in comparison with post‐mortem lesions and culture and/or nested‐PCR results.

A. (%)					B. (%)			C. (%)		
IV.	**Experimental**	**Lesion**		**Total**	**Lesion**		**Total**	**Lesion**		**Predictive value**
		**Positive**	**Negative**		**Positive**	**Negative**		**Se**	**Sp**	
	
	Positive	14	19	33	9.52	12.93	22.45	—	—	42.42
	Negative	34	80	114	23.13	54.42	77.55	—	—	70.18
	Total	48	99	147	32.65	67.35	100	29.17	80.81	—

		General agreement		63.94	Kappa agreement	—	(week)	True prevalence (Pt)		32.66

V.	**Commercial**	**Lesion**		**Total**	**Lesion**		**Total**	**Lesion**		**Predictive value**
		**Positive**	**Negative**		**Positive**	**Negative**		**Se**	**Sp**	
	
	Positive	6	9	15	4.08	6.12	10.20	—	—	40.00
	Negative	39	93	132	26.53	63.27	89.80	—	—	70.45
	Total	45	102	147	30.61	69.39	100	13.33	91.18	—

		General agreement		67.35	Kappa agreement	5.54	(week)	True prevalence (Pt)		30.60

VI.	**Experimental**	**Culture and/or nested-PCR**		**Total**	**Culture and/or nested-PCR**		**Total**	**Positive**		**Predictive value**
		**Positive**	**Negative**		**Positive**	**Negative**		**Se**	**Sp**	
	
	Positive	10	12	22	8.20	9.84	18.03	—	—	45.45
	Negative	33	67	100	27.05	54.92	81.97	—	—	67.00
	Total	43	79	122	35.25	64.75	100	23.26	84.81	—

		General agreement		63.12	Kappa agreement	9.08	(week)	True prevalence (Pt)		35.19

VII.	**Commercial**	**Culture and/or nested-PCR**		**Total**	**Culture and/or nested-PCR**		**Total**	**Positive**		**Predictive value**
		**Positive**	**Negative**		**Positive**	**Negative**		**Se**	**Sp**	
	
	Positive	3	2	5	2.46	1.64	4.10	—	—	60.0
	Negative	40	77	117	32.79	63.11	95.90	—	—	65.81
	Total	43	79	122	35.25	64.75	100	6.98	97.47	—

		General agreement		65.27	Kappa agreement	*—*	(week)	True prevalence Pt)		35.20

The commercial ELISA exhibited higher specificity in CCITT‐negative herds, while the experimental ELISA performed better in CCITT‐positive herds. When the commercial ELISA was the reference, the experimental ELISA sensitivity and specificity were 30.3% and 84.0%, respectively, again with minimal Kappa agreement (Table 1 III.).

Stratified by herd category, seroprevalence was higher in accredited herds for both ELISAs. Among negative herds, the commercial ELISA showed slightly better detection, whereas the experimental ELISA performed better in positive herds (Figure 4).

## 4. Discussion

The present study observed unexpectedly high seropositivity rates in several bTB‐accredited free herds using both the commercial and experimental ELISAs, which raises important questions about the potential causes of such reactivity. One hypothesis previously suggested is that this reactivity might be due to sensitization from repeated intradermal tuberculin (PPD) testing, especially in herds with a history of frequent skin tests. While this could be plausible in herds known to be infected and subject to repeated PPD injections, the scientific literature largely indicates that in truly tuberculosis‐free animals, PPD sensitization does not cause false‐positive serological reactions (e.g., [6, 7, 22]). Thus, this important caveat should be emphasized to avoid over‐attributing ELISA positivity to PPD sensitization alone.

Indeed, several studies, including Waters et al. [23], highlight that serological test performance can be enhanced by an anamnestic antibody response following tuberculin injection, particularly when sera are collected 1–3 weeks post‐PPD skin testing. In contrast, in our study, serum samples were collected contemporaneously with CCITT and exhibited considerable variability in the interval since the previous skin test—ranging from several months to over a year. This inconsistency challenges the evaluation of ELISA performance under ideal conditions for detecting the anamnestic response, and may partly explain the low agreement and sensitivity observed between ELISAs and CCITT.

For example, in herd I, which showed high seropositivity rates (24.26% experimental ELISA, 62.13% commercial ELISA), animals underwent annual PPD testing and previously experienced a more intensive testing regime 2 years earlier. The magnitude of seroreactivity in this herd is more consistent with residual or ongoing infection (i.e., the persistence of infected animals despite control and culling measures) rather than mere PPD sensitization, especially given the time elapsed since the last tuberculin exposure. Conversely, herds, such as A and M, which had no recent skin testing, showed very low or absent ELISA reactivity. This discrepancy strengthens the argument that infection, not PPD alone, drives high seropositivity, thereby weakening the hypothesis that PPD alone accounts for high seropositivity rates observed in herds like I.

In herds R, S, and U, strong experimental ELISA responses coincided with frequent PPD testing and commercial ELISA positivity, suggesting an interplay of active infection and humoral immune boosting possibly influenced by repeated tuberculin sensitization. Notably, herd G, despite culling of CCITT‐positive animals before serum collection, still presented seropositive individuals in the experimental ELISA, which may reflect residual infection or sustained antibody responses beyond skin test reactivity.

Taken together, these observations suggest three main contributors to seroreactivity patterns: (1) true infection with *M. bovis*, (2) immune stimulation from repeated tuberculin injections, and (3) selective removal of reactive animals through culling. Among these, infection remains the most consistent explanation, especially where seropositivity correlates with culture‐ or PCR‐confirmed *M. bovis* detection or visible lesions at slaughter.

Our data further illustrate the limitations of interpreting serological ELISA results in isolation. The incomplete overlap among tests reflects the known shift from cell‐mediated immunity (detected by CCITT) to humoral immunity (detected by ELISAs) as infection progresses to chronic or anergic stages. Despite this complexity, three main contributors to the seroreactivity patterns can be identified: (1) actual *M. bovis* infection, (2) immune stimulation from repeated tuberculin injections, and (3) selective culling of reactive animals. Among these factors, infection remains the most consistent explanation, as seropositivity frequently correlated with direct *M. bovis* detection confirmed by culture or PCR or with the presence of visible lesions at slaughter. For example, while some CCITT‐negative animals tested ELISA‐positive, they were only occasionally confirmed by molecular methods. Conversely, among CCITT‐positive animals, the experimental ELISA detected more PCR‐confirmed infections than the commercial test, providing further evidence of a possible advantage in sensitivity when detecting infected animals.

While CCITT remains a specific test for bTB, its sensitivity diminishes in late infection, limiting its value as a sole reference standard for validating antibody‐based assays. The comparative performance of CCITT in relation to culture and nested‐PCR confirmation, including sensitivity, specificity, agreement, and predictive values, is summarized in Table 3. Serological assays capture a complementary immune response and thus target different infection phases. The inclusion of ESAT‐6 in the experimental ELISA, alongside MPB70 and MPB83, may enhance detection of early or active infection stages, whereas the commercial ELISA, lacking ESAT‐6, may better identify chronic or previously exposed animals. This difference in antigenic composition likely contributes to the higher positivity of the experimental ELISA in infected herds and variable specificity.

**Table 3:** Diagnostic performance, agreement, and prevalence estimates of the comparative cervical intradermal tuberculin test (CCITT) in comparison with culture and molecular confirmation methods for *Mycobacterium bovis*.

VIII. CCITT vs. culture and/or nested‐PCR			
CCITT	Positive	Negative	Total
Positive	41	33	74
Negative	5	49	54
Total	46	82	128

**IX. CCITT + culture vs. tissue nested-PCR (TbD1)**			

**Result**	**Positive**	**Negative**	**Total**

Positive	32	3	35
Negative	49	71	120
Total	81	74	155

**X. CCITT + culture vs. culture confirmed by JB21/22**			

**Result**	**Positive**	**Negative**	**Total**

Positive	28	1	29
Negative	4	3	7
Total	32	4	36

**XI. Tissue nested-PCR (MtC) vs. culture and/or nested-PCR**			

**PCR (MtC)**	**Positive**	**Negative**	**Total**

Positive	23	11	34
Negative	11	111	122
Total	34	122	156

*Note:* General agreement: 70.31%; Kappa agreement: 43.12 (weak); true prevalence: 35.94%. General agreement: 66.45%; Kappa agreement: 34.52 (weak); true prevalence: 52.26%. General agreement: 86.11%; Kappa agreement: 47.06 (good); true prevalence: 88.89%. General agreement: 85.90%; Kappa agreement: 58.63 (good); true prevalence: 21.79%.

Abbreviation: CI, confidence interval.

The protein architecture of the experimental chimera, optimized with local sera, could also influence antigen binding and detection efficacy. However, further work is needed to characterize the immunogenicity of individual antigen fragments and their combined effects within the chimera, ideally using defined polyclonal sera for validation.

Moreover, the temporal dynamics of antigen‐specific antibody responses must be considered: MPB83 and ESAT‐6 antibodies tend to arise earlier post‐infection, while MPB70 appears later. ELISAs based on different antigen profiles will therefore capture different subsets of infected animals, partially explaining the superior sensitivity of the experimental ELISA in detecting animals missed by CCITT.

Compared with prior studies reporting higher ELISA sensitivity and specificity, our study sampled naturally infected herds with unknown infection status at testing, without artificial enrichment for infected individuals. This real‐world sampling likely contributed to wider variability in serological responses, reflecting heterogeneous infection stages, herd management, and host‐pathogen‐environment interactions—including factors such as nutrition, coinfections, and exposure to environmental mycobacteria.

Our ability to precisely evaluate ELISA diagnostic performance was limited by the absence of a true gold standard, uneven sampling of slaughtered animals, and imperfect sensitivity of culture and PCR. Particularly in low‐prevalence herds, standard ROC and frequentist measures may misrepresent test value. Hence, integrated interpretative frameworks combining serological data with epidemiological and historical herd information are essential.

In summary, serological assays, especially the experimental ELISA with broad antigenic coverage, provide a useful complement to CCITT by detecting animals potentially missed by cell‐mediated immunity tests. However, due to minimal agreement among tests and unresolved issues with sensitivity and specificity, ELISAs should not yet replace traditional diagnostics or be used alone in prevalence surveys or certification programs. Their role is better envisioned as parallel adjunct tests within comprehensive eradication strategies.

## Funding

This research was funded by the Project “Beef Cattle National Science and Technology Institute,” supported by the Brazilian National Council for Scientific and Technological Development (CNPq) (Grant 408696/2024‐9).

## Ethics Statement

To ensure confidentiality, both the veterinarians and the herds under their care remain anonymous.

## Conflicts of Interest

The authors declare no conflicts of interest.

## Data Availability

Field data supporting the results of this study were originally collected by the clinical veterinarians on routine bovine tuberculosis diagnostic tests on manuscripts by‐hand charts, copies can be found with the corresponding author and were finally transcripted to the Appendix Table 1 (pp. 78–85) of the manuscript of the doctorate thesis, all together with the ELISA results on spreadsheets on. xls format for Excell MS Office software and the consolidated data for analysis are presented in Tables 2‐5 (pp. 86–89), available with the following hyperlink to publicly archived datasets generated and analyzed inthis study: https://www.academia.edu/35231143/UNIVERSIDADE_FEDERAL_DE_SANTA_MARIA_CENTRO_DE_CI%C3%8ANCIAS_RURAIS_PROGRAMA_DE_P%C3%93S-GRADUA%C3%87%C3%83O_EM_MEDICINA_VETERIN%C3%81RIA_TESTES_DIAGN%C3%93STICOS_PARA_DETEC%C3%87%C3%83O_DE_BOVINOS_INFECTADOS_POR_Mycobacterium_bovis?email_work_card=interaction_paper.
